# Spatial computation of intratumoral T cells correlates with survival of patients with pancreatic cancer

**DOI:** 10.1038/ncomms15095

**Published:** 2017-04-27

**Authors:** Julienne L. Carstens, Pedro Correa de Sampaio, Dalu Yang, Souptik Barua, Huamin Wang, Arvind Rao, James P. Allison, Valerie S. LeBleu, Raghu Kalluri

**Affiliations:** 1 Department of Cancer Biology, Metastasis Research Center, The University of Texas MD Anderson Cancer Center, Houston, Texas 77030, USA; 2 Department of Bioinformatics and Computational Biology, The University of Texas MD Anderson Cancer Center, Houston, Texas 77030, USA; 3 Department of Electrical and Computer Engineering, Rice University, Houston, Texas 77005, USA; 4 Department of Pathology, The University of Texas MD Anderson Cancer Center, Houston, Texas 77030, USA; 5 Department of Radiation Oncology, The University of Texas MD Anderson Cancer Center, Houston, Texas 77030, USA; 6 Department of Immunology, The University of Texas MD Anderson Cancer Center, Houston, Texas 77030, USA; 7 Department of Bioengineering, Rice University, Houston, Texas 77005, USA; 8 Department of Molecular and Cellular Biology, Baylor College of Medicine, Houston, Texas 77030, USA

## Abstract

The exact nature and dynamics of pancreatic ductal adenocarcinoma (PDAC) immune composition remains largely unknown. Desmoplasia is suggested to polarize PDAC immunity. Therefore, a comprehensive evaluation of the composition and distribution of desmoplastic elements and T-cell infiltration is necessary to delineate their roles. Here we develop a novel computational imaging technology for the simultaneous evaluation of eight distinct markers, allowing for spatial analysis of distinct populations within the same section. We report a heterogeneous population of infiltrating T lymphocytes. Spatial distribution of cytotoxic T cells in proximity to cancer cells correlates with increased overall patient survival. Collagen-I and αSMA^+^ fibroblasts do not correlate with paucity in T-cell accumulation, suggesting that PDAC desmoplasia may not be a simple physical barrier. Further exploration of this technology may improve our understanding of how specific stromal composition could impact T-cell activity, with potential impact on the optimization of immune-modulatory therapies.

## Abstract (web summary)

The functional significance of T-cell infiltration in pancreatic ductal adenocarcinoma in relation to desmoplastic stroma is unclear. Here the authors develop a method to spatially resolve tumour stroma composition and find that spatial T-cell infiltration correlates with patient prognosis regardless of desmoplasia.

The immune contexture of pancreatic adenocarcinoma (PDAC) is often considered immunosuppressive in nature, with minimal antitumour T-cell infiltration^1^. However, PDAC presents with the inherent capacity to activate a T cell-mediated antitumour response^2^,^3^,^4^,^5^,^6^, and patients with PDAC possess tumour reactive memory T cells resident in their bone marrow^2^. A study has also shown that T cells are the dominant immune component found in the stroma of primary tumour samples obtained from PDAC patients^3^ and patients with higher levels of CD4^+^ and/or CD8^+^ T cells have significantly prolonged survival^4^,^5^,^6^. Nonetheless, PDAC is considered to develop an immunosuppressive microenvironment that restricts antitumour T-cell infiltration^1^,^7^,^8^. This may, in part, result from the proposed role of activated fibroblasts or myofibroblasts and the extracellular matrix in PDAC. These major constituents of PDAC desmoplasia have been hypothesized to sequester T cells away from cancer cells^5^,^9^. Recent studies in mice also suggest that focal adhesion kinase activity in cancer cells mediates an inverse correlation between fibrosis in the desmoplastic stroma and T-cell infiltration in PDAC^10^. While these mouse studies suggest that PDAC desmoplasia might act as a barrier for T-cell infiltration^5^,^9^,^10^, promising early results seen with T-cell vaccines (reviewed in ref. 8) provide evidence that T cells have the capability to infiltrate the PDAC microenvironment. Regulatory T-cell (Treg) infiltration within the PDAC stroma is observed adjacent to cancer cells, providing additional evidence for the existence of a complex regulation of T-cell infiltration as a part of the evolving PDAC desmoplasia^11^. The exact nature of the complex interaction between desmoplastic fibrotic stroma and T-cell infiltration and its impact on PDAC patient prognosis and overall survival remains to be elucidated.

The function of PDAC-infiltrating T cells may be attenuated by the co-infiltration of immune suppressive cells, such as Treg cells, or myeloid-derived suppressor cells and M2 macrophages^3^. The abundance of these cells correlates with poor tumour differentiation and/or survival in preclinical and clinical studies^3^,^12^,^13^,^14^,^15^,^16^. These observations offered support for the development of clinical efforts to target these immune cell populations using GVAX (a granulocyte-macrophage colony-stimulating factor gene-transfected tumour cell vaccine) or agonistic CD40 antibodies. The survival benefits of these strategies are lacking in PDAC preclinical models without T cells and diminished in patients with low T-cell numbers^17^,^18^. Furthermore, the antitumour efficacy of these therapies is best realized in the presence of endogenous antitumour T cells, evidenced by the combination with immune-checkpoint blockade therapies (anti-PD-1, -PD-L1 and/or –CTLA4 (cytotoxic T-lymphocyte-associated protein 4)) enhancing their antitumour efficacy^15^,^16^,^19^. These studies suggest that modulation of the immune composition in PDAC, in particular T cells, may offer clinical benefit to control and suppress PDAC progression. To harness such clinical benefit, a better understanding of the dynamic PDAC immune composition is essential.

The exploration of the microenvironmental composition of treatment-naive PDAC samples would offer critical insights into the complex and heterogeneous immune landscape associated with the growth and progression of this tumour. We thus set out to query the desmoplastic, mesenchymal and lymphocytic contexture of resected human PDAC tissue samples obtained from patients who did not receive neoadjuvant therapies. We probed formalin-fixed, paraffin-embedded (FFPE) tissue sections using a novel tyramide signal amplification (TSA) multiplexing technique to enable the simultaneous examination of eight distinct markers. The abundance and spatial organization of αSMA, Collagen-I, cytokeratin 8, CD3, CD8, CD4 and Foxp3 immunolabelled cells (nuclei labelled with 4,6-diamidino-2-phenylindole (DAPI)) were studied along with clinical features to carefully annotate the aforementioned stromal elements and their correlation with patient survival. Our study shows that distinct T-cell subpopulations infiltrate PDAC with specific spatial distributions. We also observe that stromal fibroblasts and type I collagen (Collagen-I) do not serve as absolute inhibitors of T-cell infiltration.

## Results

### Heterogeneous T lymphocyte subpopulations infiltrate PDAC

**Figure 1:**
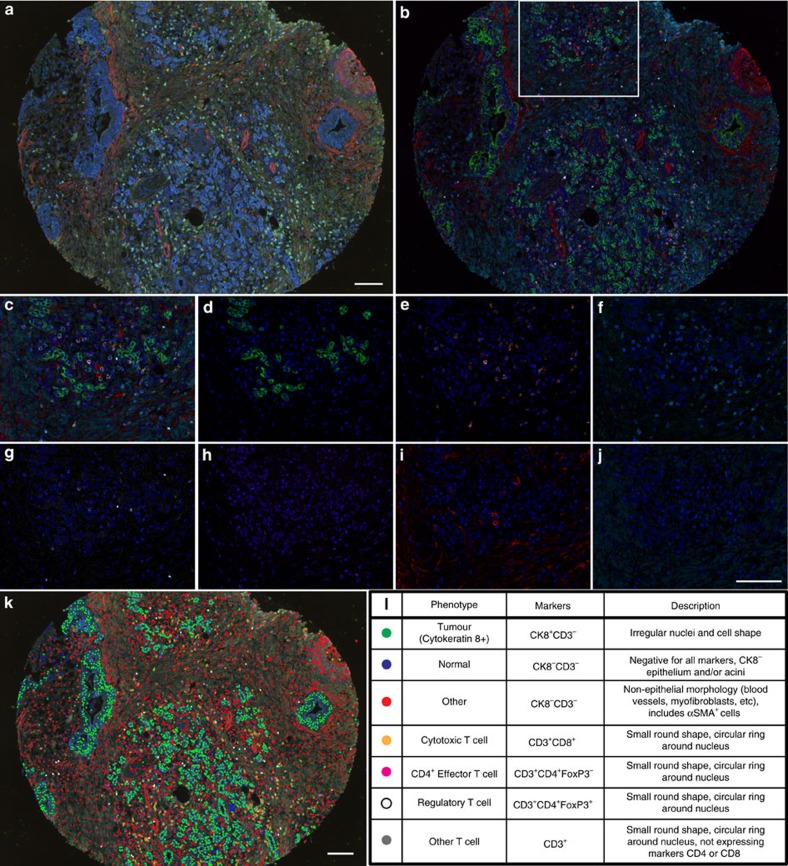
Opal eight-colour multiplex analysis of human PDAC identifies unique cellular subpopulations. (**a**,**b**) Representative images displaying the same TMA core after multispectral imaging (raw image (**a**)) and after spectral unmixing (composite image (**b**)). (**c**–**j**) Enlarged subsection of the core highlighted in **b**, showing each of the individual markers in the composite image after spectral unmixing, together with the DAPI nuclear marker (pseudocoloured blue) and the autofluorescence signal (pseudocoloured black); (**c**) all markers, (**d**) cytokeratin 8 (cytoplasmic, labelled with FITC, pseudocoloured green), (**e**) CD8 (membrane, Opal10, pseudocoloured orange), (**f**) CD3 (membrane, Opal9, pseudocoloured cyan), (**g**) Foxp3 (nuclear, Cy3, pseudocoloured white), (**h**) CD4 (membrane, Cy5, pseudocoloured magenta), (**i**) αSMA (cytoplasmic, Cy5.5, pseudocoloured red) and (**j**) Collagen-I (extracellular, Coumarin, pseudocoloured teal blue). (**k**) Cell phenotype map identifying the cell populations defined by the individual markers in the multiplex stain, overlaid on the raw image. (**l**) Summary of each defined cell phenotype, colour code and associated markers. All scale bars equal 100 μm.

**Figure 2:**
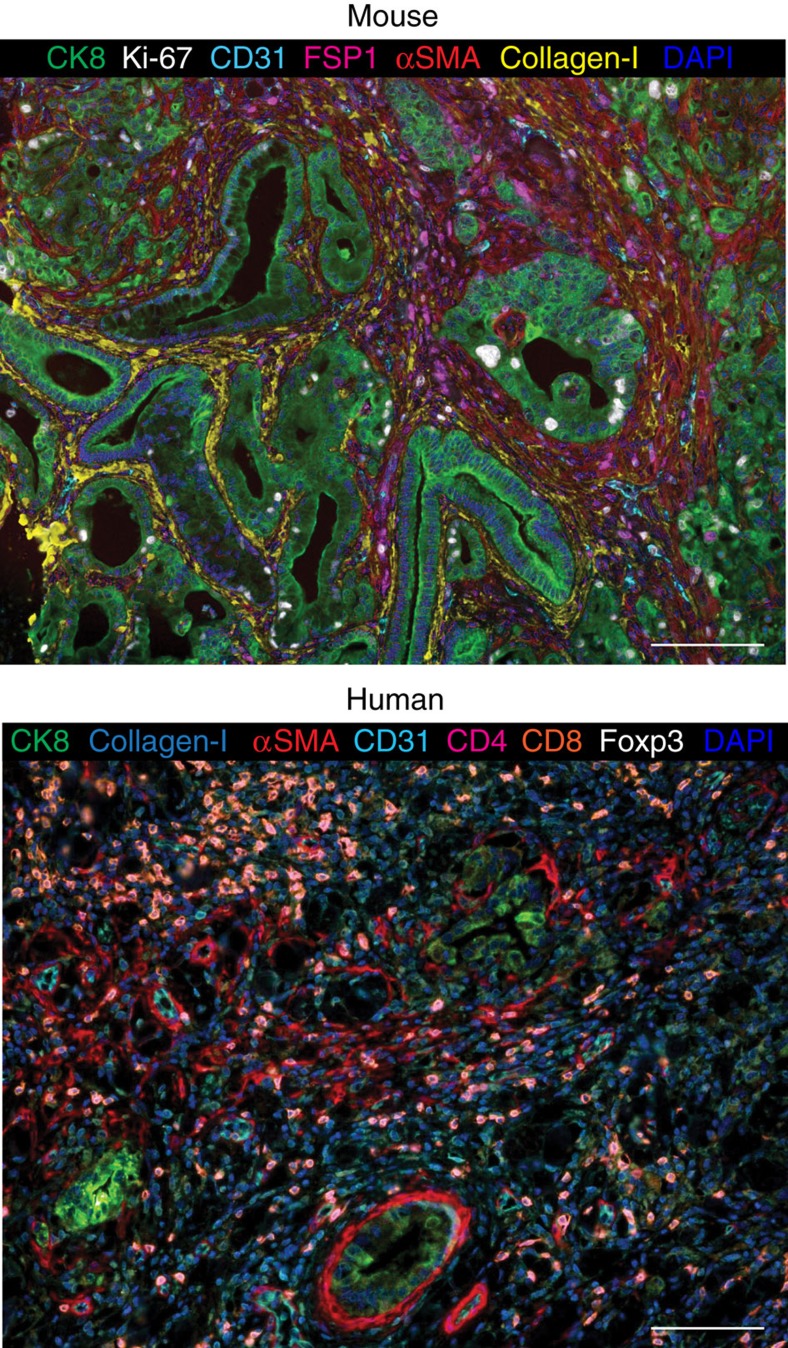
Applications of multiplex staining protocols in PDAC tissue sections. Spectrally unmixed images obtained after multispectral imaging of a mouse PDAC tumour section from a mutant Kras and mutant p53-driven PDAC mouse model and a tumour section from a PDAC patient after multiplex staining with different marker–fluorophore combinations. Mouse: cytokeratin 8 (CK8, cytoplasmic, labelled with Cy3.5, pseudocoloured green), Ki-67 (nuclear, Cy5, pseudocoloured white), CD31 (membrane, Cy3, pseudocoloured cyan), FSP1 (membrane, 680, pseudocoloured magenta), αSMA (cytoplasmic, Coumarin, pseudocoloured red), Collagen-I (extracellular, FITC, pseudocoloured yellow), autofluorescence (pseudocoloured black) and the DAPI nuclear marker (pseudocoloured blue). Human: cytokeratin 8 (CK8, cytoplasmic, Cy3.5, pseudocoloured green), Collagen-I (extracellular, Coumarin, pseudocoloured teal blue), αSMA (cytoplasmic, FITC, pseudocoloured red), CD31 (membrane, Cy3, pseudocoloured cyan), CD4 (membrane, 680, pseudocoloured magenta), CD8 (membrane, Cy5, pseudocoloured orange), Foxp3 (nuclear, Biotin-Streptavidin 594, pseudocoloured white), autofluorescence (pseudocoloured black) and the DAPI nuclear marker (pseudocoloured blue). All scale bars equal 100 μm.

We developed a novel multiplex immunolabelling protocol based on TSA, using Opal fluorophores (Supplementary Fig. 1A), which allowed for the simultaneous evaluation of eight markers in a single FFPE tissue section. Multispectral imaging was applied to the eight-marker-stained samples. This entailed the capturing of an image every 10 nm through the full emission spectrum of each filter cube (DAPI, fluorescein isothiocyanate (FITC), Cy3, Texas Red and Cy5, Supplementary Fig. 1B), which were then combined into one ‘image cube' per field of view (henceforth termed ‘raw images', Fig. 1a). A spectral signature for each fluorophore was obtained using the same multispectral imaging protocol on single stained slides, as well as a no primary control slide to obtain the autofluorescence signature of the tissue. These spectral signatures were then used to separate the raw image cube into its individual fluorophores, in a process termed spectral unmixing (Supplementary Fig. 1 and Fig. 1b–j). We used this technology to probe human and mouse PDAC tissue samples for multiple combinations of stromal markers (Fig. 1 and Fig. 2). As anticipated, a complex and heterogeneous tumour stroma was noted, which included mesenchymal and T lymphocytic components with varying abundance and distribution (Fig. 1 and Fig. 2). In order to study the infiltration of different T-cell subpopulations in PDAC and their potential interactions with the mesenchymal stroma, we focussed on the following set of markers: alpha-smooth muscle actin (αSMA), Collagen-I, cytokeratin 8, CD3, CD8, CD4, Foxp3, and DAPI (nuclear stain) (Fig. 1 and Supplementary Fig. 1). Multispectral imaging followed by spectral unmixing allowed for the simultaneous evaluation of all markers in each tissue sample (Fig. 1b–j). A comparison of the unmixed images from multiplex stained tissues with tissue sections individually stained for each marker demonstrated the efficacy of the spectral unmixing algorithm (Supplementary Fig. 1C). The spectrally unmixed images were then analysed to identify different cellular phenotypes, based on the aforementioned markers as well as cellular size and morphology (phenotyping) (Fig. 1k,l). The marker CD3, in addition to the shape of the nucleus, was used to identify all T cells. Subpopulations of T cells were then defined by the presence and absence of three additional markers: CD8, CD4, and Foxp3. For the purpose of this study, cytotoxic T cells were defined as CD3^+^CD8^+^CD4^−^Foxp3^−^, CD4^+^ effector T cells (CD4^+^ Teff) as CD3^+^CD8^−^CD4^+^Foxp3^−^, Treg cells as CD3^+^CD8^−^CD4^+^Foxp3^+^ and any CD3^+^ cells negative for the three other markers were defined as ‘other T cells'. Cytokeratin 8 was used to identify epithelial cancer cells in tumour samples and benign pancreatic ductal cells in uninvolved tissue samples. Pancreatic acinar cells were observed in the uninvolved pancreatic tissue and in a small percentage of tumour tissue. These cells were negative for all markers but were distinguishable from other populations based on morphology, as detected through the autofluorescence signal. We defined this population as ‘normal', to reflect their non-transformed histological nature. Finally, all other cells not defined in our phenotyping categories (that is, myofibroblasts, blood vessels, nerves, pancreatic islets, macrophages and so on) were grouped into one category, labelled as ‘other'. Representative images of all analysed cellular phenotypes are shown in Supplementary Fig. 2.

**Figure 3:**
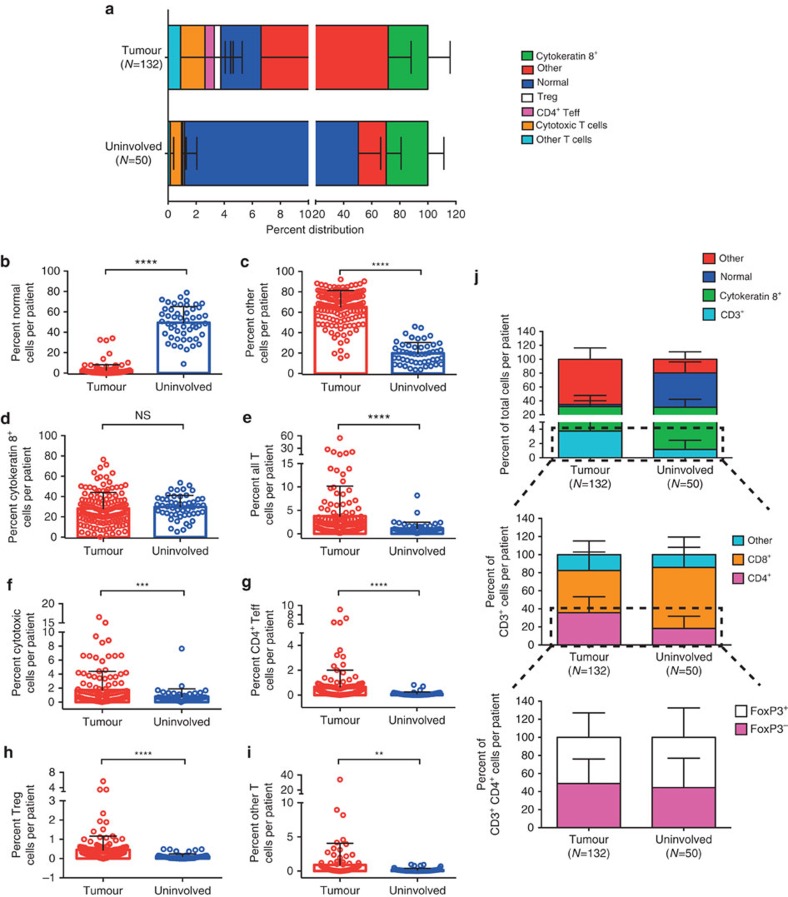
PDAC tissue samples display increased infiltration of heterogeneous T-cell subpopulations. (**a**) Relative distribution of all analysed cell phenotypes in PDAC and uninvolved pancreatic tissue samples. (**b**–**i**) Pairwise comparisons of the percentage of cells per patient for normal cells (**b**), other cells (**c**), cytokeratin 8^+^ cells (**d**), all T cells (**e**), cytotoxic T cells (**f**), CD4^+^ Teff cells (**g**), Treg cells (**h**) and other T cells (**i**) between tumour and uninvolved pancreatic tissue samples. Significance determined by unpaired *t*-test. (**j**) Relative distribution analysis of different T-cell phenotypes within defined groups, by separating the total cell number initially into other, normal, cytokeratin 8^+^ cells or total CD3^+^ T cells (includes all T-cell subpopulations; upper panel); then focussing only on CD3^+^ T cells and dividing them into CD4^+^ (helper T cells), CD8^+^ (cytotoxic T cells) and CD4^−^CD8^−^ (other T cells) subpopulations (middle panel); and finally focussing on CD3^+^CD4^+^ T cells and dividing them into FoxP3^+^ (Tregs) and FoxP3^−^ (CD4^+^ Teff) subpopulations (lower panel). Data presented as the mean±s.d. **P<0.01, ***P<0.001, ****P<0.0001, NS, not significant.

Tissue microarrays (TMAs) comprised of tumour and uninvolved tissue samples obtained from 132 PDAC patients were used for phenotyping (Supplementary Fig. 3). Whenever available, two TMA tumour tissue cores collected from different FFPE blocks were analysed and the combined percentage of cell counts were calculated per patient. Cores were only excluded if no analysable tissue were present and not on the account of heterogeneity between the cores, as is common, in order to capture the heterogeneity of the tumour. The clinical characteristics of the patients represented in the TMAs are detailed in Table 1. Of note, uninvolved pancreatic tissue specimens of good quality and absent of tumour tissue were only available for 50 patients. Direct comparisons between the uninvolved pancreatic tissue and tumour specimens for these 50 patients are presented in Supplementary Fig. 4A–I. When all available cancer and uninvolved cores were taken into account, we observed the tumour samples presented with significantly less normal cells than the uninvolved tissue (Fig. 3a,b). ‘Other' components, as defined above, which include αSMA^+^ myofibroblasts and associated desmoplasia, were also more abundant in the tumour tissue as compared to the uninvolved pancreatic tissue (Fig. 3a,c). Interestingly, the uninvolved pancreatic tissue samples contained significant amounts of ductal and acinar cells that expressed cytokeratin 8 (Fig. 3a,d, Supplementary Fig. 4J,K). This resulted in no significant differences in the percentage of cytokeratin 8^+^ cells between tumour and uninvolved pancreatic tissues (Fig. 3d). These adjacent uninvolved pancreatic tissue cores, as opposed to fully normal samples, may have exhibited chronic pancreatitis and early stages of transformation (that is, acinar to ductal metaplasia) that could be reflected by their expression of cytokeratin 8. Finally, the total number of T cells, as well as the numbers of all T-cell subpopulations analysed, were higher in the tumour tissue when directly compared to the uninvolved tissue (Fig. 3a,e–i).

When we divided all CD3^+^ cells into the different T-cell subpopulations, we observed that >80% of the CD3^+^ T cells were either CD4^+^ or CD8^+^ in both tumour and uninvolved pancreatic tissues (Fig. 3j, middle panel). Significantly fewer CD3^+^ T cells were noted in the uninvolved pancreatic tissue compared to the tumour tissue (Fig. 3e), yet the relative proportion of CD8^+^ T cells within total CD3^+^ cells was greater in uninvolved (67%) compared to tumour (47%, *P* value <0.001) and the relative proportion of CD4^+^ within total CD3^+^ cells was lower in uninvolved (17%) compared to tumour tissue (36%, *P* value <0.001) (Fig. 3j, middle panel). The higher proportion of cytotoxic T cells within total CD3^+^ T cells in the uninvolved tissue may reflect an active immune reaction against the abundant cytokeratin 8^+^ cells, which could represent initial stages of cancer cell transformation. Alternatively, the high number of cytotoxic T cells in the uninvolved tissue might represent a reaction to the presence of the tumour. Among CD4^+^ T cells, both CD4^+^ Teffs (CD4^+^ FoxP3^−^) and Tregs (CD4^+^ FoxP3^+^) infiltrated the tumour and uninvolved pancreatic tissue in equal relative proportions in both tumours and uninvolved tissue (Fig. 3j, lower panel). Collectively, these results indicated that a significantly more abundant CD3^+^ T-cell infiltration is found in tumours when compared to uninvolved pancreatic tissue. These tumour-infiltrating T-cell populations are mostly comprised of CD8^+^ cytotoxic T cells and CD4^+^ helper T cells (CD4^+^ Teff and Treg).

### T-cell infiltration correlates with PDAC patient survival

**Figure 4:**
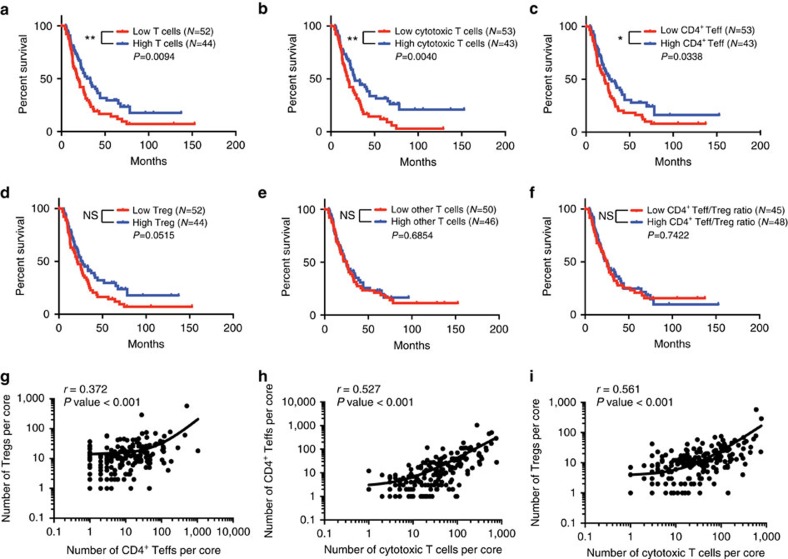
T-cell infiltration significantly stratifies patient survival. (**a**–**f**) Survival analysis of all 132 patients based on percentage of cell numbers per patient of all CD3^+^ T cells (**a**), CD3^+^CD8^+^ cytotoxic T cells (**b**), CD3^+^CD4^+^Foxp3^−^ Teffs (**c**), CD3^+^CD4^+^FoxP3^+^ Tregs (**d**), CD3^+^CD4^−^CD8^−^ other T cells (**e**) and the ratio of CD4^+^ Teff/Treg per patient (**f**). *N* values correspond to uncensored patients (who reached cancer survival end point). In **f**, patients with no Tregs were excluded (*N*=3 patients excluded) as no ratio could be calculated. High and low infiltration values were divided based on the median percentage of positive cells or ratio. Significance was determined using the Log-rank Mantel–Cox test. (**g**–**i**) Correlation analysis between Treg and CD4^+^ Teff cell counts (**g**), cytotoxic T cells and CD4^+^ Teff cell counts (**h**) and cytotoxic T cells and Treg cell counts (**i**) per core. Pearson correlation coefficient (*r*) and significance levels (*P* value) are presented for each correlation. Axis values are shown in log scale for clarity. *P<0.05, **P<0.01, NS, not significant.

We next sought to define whether specific T-cell infiltration in PDAC independently correlated with patient survival. We computed the percentage of T-cell subpopulations out of the total number of cells for each patient, calculating the combined cell percentages in both cores when multiple cores where available per patient. Patients were stratified into low or high tumour-infiltration groups based upon the median percentages for each T-cell subpopulation. We observed that high levels of total T-cell infiltration were associated with prolonged survival (Fig. 4a). This highlights the clinical relevance and possible functional importance of T-cell infiltration for PDAC progression. High infiltration of cytotoxic T cells and CD4^+^ Teff cells also correlated with a significant increase in patient survival (Fig. 4b,c), whereas infiltration of Treg or other T cells did not significantly associate with survival (Fig. 4d,e). In light of the opposing functions of CD4^+^ Teffs and Tregs, we examined the ratio of CD4^+^ Teff/Treg cells but observed no association with outcome in this cohort of patients (Fig. 4f). We also observed a positive correlation between the three T-cell subpopulations (Fig. 4g–i), inferring that Tregs are increased when there is an increase in CD4^+^ Teff or cytotoxic T cells, possibly accounting for the lack of differential infiltration pattern with predictive survival value for Tregs. Altogether these results suggested that CD4^+^ Teff and cytotoxic T cells are defining determinants of patient survival in PDAC.

Beyond their association with survival, infiltration levels of total T cells did not significantly associate with any other clinical parameters (Supplementary Table 1). This was also true for the infiltration levels of all T-cell subpopulations (Supplementary Tables 2–6). In the cohort studied here, a subset of measured clinical parameters also had a significant impact on survival, namely, surgical margins, lymph node status and adjuvant chemotherapy (Kaplan–Meier/univariate analysis, Table 2). We therefore investigated whether the infiltration levels of the T-cell subpopulations shown to associate with survival did so independently of these factors. A stepwise multivariate Cox regression analysis was performed comparing each of the T-cell populations with the clinical features listed above. We observed that total T-cell infiltration (*P* value =0.014), cytotoxic T-cell infiltration (*P* value=0.002) and CD4^+^ Teff infiltration (*P* value=0.032) all maintained an independent association with survival even in the presence of varying surgical margins, lymph node positivity or adjuvant chemotherapy (Table 2).

### Cancer cell-adjacent cytotoxic T cells correlate with survival

**Figure 5:**
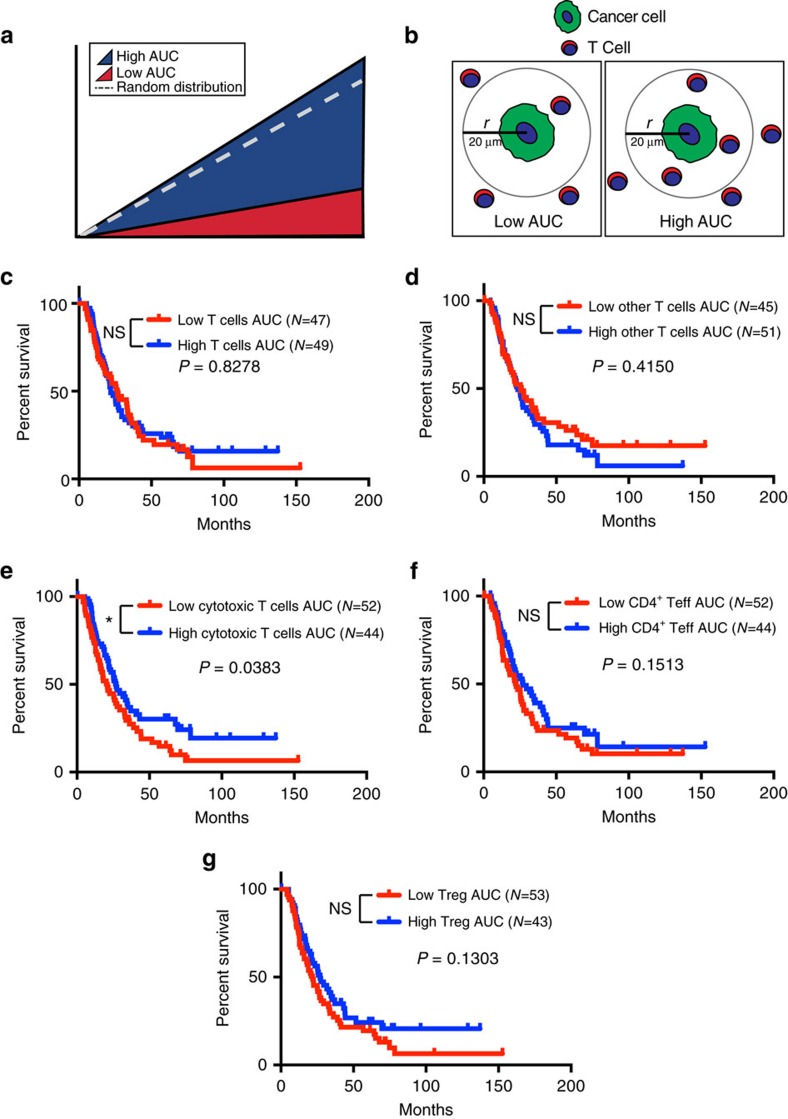
Cancer cell-adjacent cytotoxic T cells significantly correlate with survival. (**a**) Schematic representation of the AUC levels based on an L-function calculated for T-cell infiltration. Dashed line—random distribution, Blue area—high AUC levels/High infiltration, Red area—low AUC levels/low infiltration. (**b**) Schematic representing the calculation of the L-function based on the distribution of T cells within a radius of 20 μm from the nuclear centre of a cytokeratin 8^+^ cancer cell. (**c**–**g**) Survival analysis of all 132 patients based on infiltration as determined by the AUC levels of all CD3^+^ T cells (**c**), CD3^+^CD4^−^CD8^−^ other T cells (**d**), CD3^+^CD8^+^ cytotoxic T cells (**e**), CD3^+^CD4^+^Foxp3^−^ Teffs (**f**) and CD3^+^CD4^+^FoxP3^+^ Tregs (**g**) within 20 μm of cytokeratin 8^+^ cancer cells. *N* values correspond to uncensored patients (who reached cancer survival end point). High and low infiltration values were calculated using the L-function AUC values and divided based on the median infiltration values. Significance was determined using the Log-rank Mantel–Cox test. *P<0.05, NS, not significant.

To explore whether spatial distributions of intratumoral T cells with respect to cancer cells correlate with patient outcome, we determined the spatial coordinates of each T-cell subpopulation. These coordinates were used to characterize the spatial point patterns of T cells relative to cytokeratin 8^+^ cancer cells, using Ripley's L-function^20^,^21^. This methodology was previously used to demonstrate a prognostic value of heterogeneous cellular spatial patterns in breast cancer using hematoxylin and eosin-stained tissue specimens^22^,^23^. An L-function is defined such that, in this case, the number of T cells within a specified radius (*r*) distributed from a given point (nuclear centres of cytokeratin 8^+^ cancer cells) is 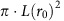. If the T-cell population is randomly distributed relative to the cancer cells, the underlying theoretical L-function will have the form *L*(*r*)=*r*, represented by a linear slope (Fig. 5a and Supplementary Fig. 5, dashed line). The area under the L-function curve (AUC) can therefore be used to measure the infiltration of T cells within a specified radius around the cancer cells (Fig. 5a and Supplementary Fig. 5). Low T-cell infiltration into the tumour will correspond to a low AUC value (Fig. 5a, red), whereas high T-cell infiltration will correspond to a high AUC value (Fig. 5a, blue). This is further exemplified in Supplementary Fig. 5, where patients A–C have increasing levels of cytotoxic T-cell infiltration in relation to cancer cells, corresponding to increasing AUC levels. We focussed on a 20 μm radius around the cancer cells, which represents an enhanced probability for cell–cell contact (Fig. 5b). The AUC values were calculated for each T-cell subpopulation (all T cells, cytotoxic T cells, CD4^+^ Teff, Treg and other T cells) within a 20 μm radius of cytokeratin 8^+^ cancer cells. Our results showed that the infiltration levels of most T-cell subpopulations within this radius did not significantly associate with survival (Fig. 5c–g). However, high infiltration of cytotoxic T cells, within a 20 μm radius of cytokeratin 8^+^ cancer cells, significantly correlated with prolonged patient survival (Fig. 5e). This suggests that cytotoxic T cells within the direct vicinity of cancer cells may perform an important biological function. This is in accordance with the required cell-cell contact necessary for cytotoxic T cells' antitumour activity^24^.

### Desmoplastic elements do not limit lymphocytic infiltration

**Figure 6:**
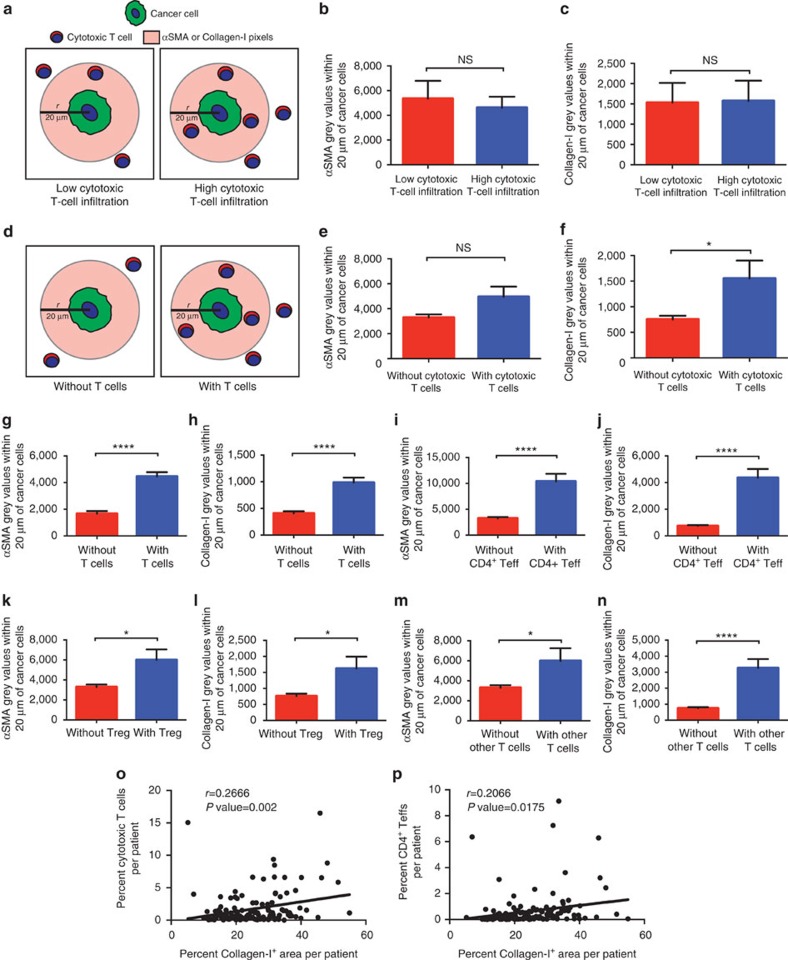
Desmoplastic elements associate with T-cell infiltration. (**a**) Schematic representing the parameters analysed in **b**,**c**. (**b**,**c**) The mean intensity of αSMA (**b**) and Collagen-I (**c**) (grey values) for pixels within 20 μm of cancer cells for each patient separated by low or high cytotoxic T-cell infiltration as determined by the L-function AUC; significance determined by an unpaired *t*-test. (**d**) Schematic representing the parameters analysed in **e**–**n**. (**e**–**n**) The mean intensity of αSMA (**e**,**g**,**i**,**k**,**m**) and Collagen-I (**f**,**h**,**j**,**l**,**n**) (grey values) for pixels within 20 μm of cancer cells for each patient separated by cancer cells with or without adjacent cytotoxic T cells (**e**,**f**), all T cells (**g**,**h**), CD4^+^ Teffs (**i**,**j**), Tregs (**k**,**l**) and other T cells (**m**,**n**); significance determined by an unpaired *t*-test. (**o**,**p**) Correlation analysis between area of Collagen-I deposition and the percentage of cytotoxic T cells (**o**) and area of Collagen-I deposition and the percentage of CD4^+^ Teffs (**p**) per patient. Pearson correlation coefficient (*r*) and significance levels (*P* value) are presented for each correlation. Data presented as the mean±s.e.m. *P<0.05, ****P<0.0001, NS, not significant.

The dense desmoplasia surrounding PDAC has been proposed to impede lymphocyte infiltration^5^,^9^. Therefore, we investigated whether infiltration of cytotoxic T cells adjacent to cancer cells was associated with reduced levels of fibrotic stroma. The fluorescent pixel intensity within a 20 μm radius of cytokeratin 8^+^ cancer cells was determined for both αSMA and Collagen-I (Fig. 6a). Unspecific background fluorescence levels were determined using unstained tissue within the image and were subtracted from αSMA or Collagen-I component images. The remaining intensity level (in grey counts), representing αSMA or Collagen-I fluorescent pixel intensity, was determined for each pixel within the 20 μm radius of each cancer cell (Fig. 6a). In patients stratified by the AUC levels of cytotoxic T cells within the same radius (high versus low levels of cancer cell-adjacent cytotoxic T cells), the αSMA or Collagen-I intensities were similar (Fig. 6b,c), suggesting that varying levels of αSMA or Collagen-I did not affect cytotoxic T-cell infiltration. These results challenge the postulated role of αSMA^+^ cells or Collagen-I content as negative regulators of T-cell infiltration^5^,^9^. We next compared αSMA or Collagen-I content adjacent to cancer cells with and without cytotoxic T cells' infiltration (Fig. 6d). A similar result was obtained with respect to αSMA intensity levels: cancer cells in close contact with cytotoxic T cells showed insignificant difference (*P* value=0.0539) in pericellular αSMA expression compared to cancer cells with no cytotoxic T-cell infiltration (Fig. 6e). In contrast, pericancer cell areas containing cytotoxic T cells had significantly higher Collagen-I content (Fig. 6f). We additionally compared the αSMA or Collagen-I content adjacent to cancer cells with or without each of the remaining pericellular T-cell subpopulations and noted that all T cells combined, CD4^+^ Teffs, Tregs and other T-cell subpopulations were found adjacent to cancer cells associated with higher levels of either αSMA or Collagen-I (Fig. 6g–n). We finally looked at the correlation between the expression of markers of desmoplasia and T-cell infiltration in the full context of the TMA cores. While αSMA levels did not significantly correlate with T-cell infiltration (Supplementary Fig. 6), we observed a positive correlation between Collagen-I deposition and the percentage of tissue-infiltrating cytotoxic T cells and CD4^+^ Teff (Fig. 6o,p). These results demonstrated the relationships between T lymphocytes and cellular (myofibroblasts) and non-cellular (Collagen-I) desmoplasia were more heterogeneous than had been previously appreciated. Our results indicated that a fibrotic reaction in the PDAC microenvironment may not impair the infiltration of T cells and that increased levels of Collagen-I deposition rather appear to correlate with the presence of different T-cell subpopulations. Future detailed mechanistic exploration is, however, required.

## Discussion

The precise nature of PDAC immunity requires a comprehensive understanding of its microenvironmental complexities. Spatial relationships of individual cellular and acellular components in PDAC may offer novel insights into the dynamic and complex functions of PDAC desmoplasia. Here we have established a novel computational methodology to probe the spatial features of both mesenchymal and T lymphocytic components in the PDAC stroma. This technology benefits from the combined bioinformatics power of flow cytometry, with its use of several combined markers, and the spatial information obtained from immunohistochemistry. The use of multiple markers is important as it allows for the identification of distinct subpopulations in one tissue section. For instance, Foxp3, commonly used to identify Tregs^5^,^25^, is also expressed in cancer cells^26^, therefore, the use of just this one marker could overestimate the abundance of tumour-infiltrating Tregs. Additionally, the spatial relationships between T-cell subpopulations and cancer cells are retained in our experimental strategy, allowing for a more precise appreciation of their biological interactions.

We applied this technology to a TMA comprised of tissue obtained upon pancreatectomy of 132 patients with PDAC without neoadjuvant therapy. Two tumour cores as well as a core with non-involved tissue were available for each patient. In order to elucidate the complex nature of PDAC immunity, we utilized this technology to demonstrate that human PDAC contains a heterogeneous T-cell population. Among these, cytotoxic T cells were the predominant T-cell subpopulation (approximately 47% of all T cells) and high infiltration of cytotoxic T cells was associated with prolonged survival. Additionally, tumour samples showed a significant increase in the proportion of CD4^+^ T helper cells when compared to normal tissue, suggesting the activation of a systemic immune response to disease progression. Our data suggested that the distribution and relative abundance of tumour-promoting Tregs may not influence patient outcome to the extent that has been suggested^1^,^6^,^27^, as Treg-infiltration levels did not significantly associate with patient survival in our analysis. Interestingly, the number of PDAC-infiltrating Tregs in our study (average of 25 cells mm^−2^) was equivalent to the numbers of Tregs reported in melanoma (∼20 cells mm^−2^) assayed using similar techniques^28^. Considering melanoma is widely regarded as an immunogenic cancer, our results suggest the number of infiltrating Tregs may not be a significant determinant of PDAC low immunogenicity. In contrast, CD4^+^ Teff and cytotoxic T-cell infiltration emerged as independent indicators of survival for PDAC patients. Additionally, the distribution of cytotoxic T cells was particularly significant when found in the direct vicinity of cancer cells. These observations were performed on all evaluable PDAC tissue areas, thereby providing an overall snapshot of the PDAC T lymphocytic landscape. This also removed the bias of previous observations, which focussed only on high areas of immune infiltration for T-cell counts using single marker immunohistochemistry^4^,^6^. When both tumour cores were available for a patient, both were included for evaluation. Our analysis further supports the published data that PDAC with high levels of cytotoxic T cells have prolonged survival. It should be noted, however, that while the overall infiltration of these cells had a significant impact on survival, their functional status was not measured and remains to be explored. Combining our panel with additional multiplex panels for markers of T-cell activation, as well as markers of additional components of the PDAC microenvironment, such as myeloid cells, may elucidate the differences between PDAC and melanoma and their immunotherapy responsiveness.

The desmoplastic stroma has been hypothesized to sequester T cells away from cancer cells^5^,^9^. The multiplex technology provided us an opportunity to address this hypothesis. Here we show tumour cells with high or low pericellular cytotoxic T-cell infiltration did not exhibit differences in the associated levels of αSMA and Collagen-I. This suggested that the desmoplastic reaction as determined by αSMA and Collagen-I deposition may have insignificant impact on the infiltration of cytotoxic T cells. Recent observations have suggested that focal adhesion kinase signalling in cancer cells can mediate a fibrotic reaction with immunosuppressive consequences^10^. This was shown only in mouse models of PDAC using generic measurements of desmoplasia without spatial analysis. Using patient samples, we show that the desmoplastic reaction in the direct vicinity of cancer cells does not negatively correlate with T-cell infiltration. We further demonstrate that enhanced Collagen-I levels correlated with increased levels of cytotoxic T-cell and CD4^+^ Teff infiltration. Cancer cells devoid of pericellular cytotoxic T cells exhibited diminished Collagen-I in their vicinity, suggesting that Collagen-I deposition and remodelling may favour, rather than hinder, cytotoxic T-cell infiltration adjacent to cancer cells.

The efficacy of single-agent immune checkpoint blockade in the treatment of PDAC patients has so far been underwhelming^29^, contributing to the perception that PDAC is a poorly immunogenic tumour. Notably, in the treatment of melanoma using immunotherapy, patients with programmed death ligand 1 (PD-L1) expression in their tumours had the most beneficial response to anti-PD-L1 therapy^30^,^31^,^32^. These studies are prompting ongoing efforts to stratify patients according to defined biomarkers (that is, PD-1, PD-L1 expression or cytotoxic T cells)^30^. It is conceivable that stratification of PDAC patients could also enable the realization of significant benefit with immunotherapy. Our collective analyses suggest that such stratification in PDAC may require a detailed audit of the tumour microenvironmental components due to the complex and dense composition of mesenchymal cells and immune cells. We propose that tissue-typing the microenvironmental composition of PDAC (that is, the number of cytotoxic or helper T cells and desmoplastic stroma) may aid in defining patient populations that would most benefit from immune therapies, as proposed previously^14^. This is in accordance with growing consensus that T lymphocytic infiltration should be included in standard tumour pathological scoring^33^. In addition to measures of relative abundance, the spatial distribution analysis we report could also aid in retrospective evaluation of responses to therapy. In summary, our current observations suggest that T cells infiltrate PDAC tumours and may not be impeded by αSMA or Collagen-I stromal deposition. This study offers new insights into the nature of PDAC immunity and how this information can be harnessed towards effective immunotherapy strategies.

## Methods

### Patient cohorts

This study was approved by the institutional review board at the University of Texas MD Anderson Cancer Center (MDACC). Our study population consisted of 132 patients with PDAC who underwent pancreatectomy with curative intent (Table 1) at MDACC. Informed consent was obtained from all patients. None of the patients received neoadjuvant therapy. The PDAC TMAs were constructed from FFPE blocks of archived PDAC specimens using the method described previously^34^. Briefly, representative areas of tumour and matched uninvolved pancreatic tissue were selected based on the review of hematoxylin and eosin-stained slides. The corresponding FFPE tissue blocks were retrieved. For each patient, two 1.0 mm cores from representative areas of the tumour and one 1.0 mm core of matched uninvolved pancreas were used for TMA construction^35^. Clinical information was obtained from the electronic medical records.

### Samples for trial multiplex stains

Sections from full FFPE blocks that were used to generate the TMA were utilized in the initial optimizations of the multiplex tissues, as presented in Fig. 2. Sections of mouse PDAC samples (end point *Pdx1- cre;LSL-Kras*^*G12D*^*;P53*^*R172H/+*^ tumours) that were obtained from archived blocks from previously published work^14^ were also used in initial multiplex optimizations as presented in Fig. 2.

### Eight-colour immunohistochemical multiplex

In all, 5 μm sections obtained from the TMA blocks were deparaffinized and tissues were fixed with formaldehyde:methanol (1:10) prior to antigen retrieval in heated Citric Acid Buffer (pH 6.0) for 15 min (EZ Retriever microwave, BioGenex). Each section was put through seven sequential rounds of staining, each including a protein block with 1% BSA followed by primary antibody and corresponding secondary horseradish peroxidase-conjugated polymer (Table 3 and Supplementary Fig. 1A). Each horseradish peroxidase-conjugated polymer mediated the covalent binding of a different fluorophore using tyramide signal amplification (Table 3 and Supplementary Fig. 1A). This covalent reaction was followed by additional antigen retrieval in heated Citric Acid Buffer (pH 6.0) for 15 min to remove bound antibodies before the next step in the sequence. After all seven sequential reactions, sections were counterstained with DAPI (Life Tech) and mounted with Vectashield fluorescence mounting medium (Vector Labs, Burlingame, CA).

### Multispectral imaging

Multiplex stained TMA slides were imaged using the Vectra Multispectral Imaging System version 2 (Perkin Elmer), where one raw image comprising four stitched 200 × multispectral image cubes was obtained for each TMA core. Each 200 × multispectral image cube was created by combining images obtained every 10 nm of the emission light spectrum across the range of each emission filter cube. Filter cubes used for multispectral imaging were DAPI (440–680 nm), FITC (520 nm-680 nm), Cy3 (570–690 nm), Texas Red (580–700 nm) and Cy5 (670–720 nm) (Supplementary Fig. 1B).

### Spectral unmixing and phenotyping

A spectral library containing the emitting spectral peaks of all fluorophores was created with the Nuance Image Analysis software (Perkin Elmer) (Supplementary Fig. 1B), using multispectral images obtained from single stained slides for each marker and associated fluorophore. This spectral library was then used to separate each multispectral image cube into its individual components (spectral unmixing) allowing for the colour-based identification of all eight markers of interest in a single image using the inForm 2.1 image analysis software. All spectrally unmixed and segmented images were subsequently subjected to a proprietary inForm active learning phenotyping algorithm. This allows for the individual identification of each DAPI-stained cell according to their pattern of fluorophore expression and nuclear/cell morphological features, associating their phenotype with specific *x*,*y* spatial coordinates. Cells were phenotyped into one of seven different classes according to our markers of interest (Fig. 1l) as follows: cancer/cytokeratin 8^+^ cells (CK8^+^), cytotoxic T cells (CD3^+^CD8^+^), CD4^+^ Teff cells (CD3^+^CD4^+^Foxp3^−^), Treg cells (CD3^+^CD4^+^FoxP3^+^), other T cells (CD3^+^CD4^−^CD8^−^), normal (CK8^−^CD3^−^ and epithelial or acinar cell morphology as determined through the autofluorescence signal), and other (CK8^−^CD3^−^). All phenotyping and subsequent quantifications were performed blinded to the sample identity and clinical outcomes.

### Quantification of T-cell spatial distribution

In each spectrally unmixed and phenotyped core, the relative spatial distribution of cancer cells and each individual subpopulation of T cells were considered as a bivariate point pattern. This bivariate point pattern can be characterized by bivariate K- and L-functions, generalized from Ripley's K- and L-functions^21^. The bivariate K-function in our application is defined as follows:


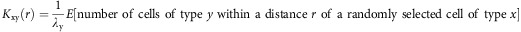


where *λ*_y_ is the number of type *y* cells per unit area in the region of interest and *E*[.] evaluates the expected value of the quantity in the bracket. Theoretically, if the spatial distribution of the two types of cells *x* and *y* are completely independent (Poisson hypothesis), the value of K-function is 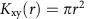 (ref. 36). The bivariate L-function is a transformation of K-function, defined as:


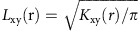


Under the Poisson hypothesis, we have 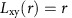, which is a more convenient representation than the K-function. Also, the estimation of 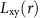 has a more stabilized variance^36^. We used the toolbox ‘spatstat' in R for the estimation of the L-function^37^. For each of the TMA images, the L-function was estimated for a range of *r* from 0 to 20 μm. The level of infiltration of different T cells is represented by the AUC of their L-function and dichotomized at the median to discriminate patient survival.

### Quantification of fluorescent pixel intensity

Monochromatic tiff images of the spectrally unmixed cores containing only the Cy5.5 (αSMA) or Coumarin (Collagen-I) signal components (processed separately) were analysed by ImageJ to determine the background pixel intensity levels (grey values), as determined by the unstained tissue areas. Absolute intensity levels of all pixels within 20 μm of each CK8^+^ cancer cell were determined and background intensity levels were subtracted using Matlab. Percentage of positive area for the whole core was determined by setting the positivity threshold on ImageJ using the Huang algorithm (αSMA 20/255, or Collagen-I 35/255). This positivity is represented as the percentage of all signal areas calculated from the multiplexed composite image displaying all channels, including autofluoresence (region of interest selected from all pixels over the brightness threshold of 30/255).

### Statistics

All statistical analyses were performed using the GraphPad Prism software unless stated otherwise. Statistical analyses of immunohistochemical quantifications were performed using a Student's *t*-test or analysis of variance as appropriate. For survival analyses, Kaplan–Meier plots were drawn and statistical differences were evaluated using the log-rank Mantel–Cox test (a.k.a univariate). Multivariate analyses of the survival data were performed for each new T-cell infiltration parameter using a Cox regression analysis in SPSS. For the correlation analyses between different T-cell distributions, the Pearson's correlation coefficient (*r*) was calculated using the SPSS statistical software. Pairwise comparisons between clinical variables and all survival stratifications were determined by a chi-square analysis using SPSS. A *P* value <0.05 was considered statistically significant.

### Data availability

The authors declare that the data supporting the findings of this study are available within the paper and its Supplementary Information files (Supplementary Data 1–8). All computer codes used for spatial distribution analyses as well as any additional clarifications are available from the corresponding author upon request.

## Additional information

**How to cite this article:** Carstens, J. L. *et al*. Spatial computation of intratumoral T cells correlates with survival of patients with pancreatic cancer. *Nat. Commun.* **8,** 15095 doi: 10.1038/ncomms15095 (2017).

**Publisher's note:** Springer Nature remains neutral with regard to jurisdictional claims in published maps and institutional affiliations.

## Supplementary Material

Supplementary Information Supplementary Figures and Supplementary Tables

Supplementary Data 1 Source data pertaining to Figure 3A-J. The values and statistics used to generate each panel are separated into individual tabs of the spreadsheet. Note: Each line represents the values per patient.

Supplementary Data 2 Source data pertaining to Figure 4A-I. The values and statistics used to generate each panel are separated into individual tabs of the spreadsheet. Note: Each line represents the values per patient unless indicated otherwise.

Supplementary Data 3 Source data pertaining to Figure 5C-G. Each tab of the spreadsheet contains the values and statistics used to generate each of the survival panels in the figure. Note: Each line represents the values per patient.

Supplementary Data 4 Source data pertaining to Figure 6B-C and E-P. Each tab of the spreadsheet contains the values and statistics used to generate the indicated panels in the figure. Note: Each line represents the values per patient.

Supplementary Data 5 Source data pertaining to Tables 1 and 2. Each tab of the spreadsheet contains the values and statistics used to generate each table.

Supplementary Data 6 Source data pertaining to Supplementary Figure 4A-I. Each tab of the spreadsheet contains the values and statistics used to generate each panel in the figure. Note: Each line represents the values per patient.

Supplementary Data 7 Source data pertaining to Supplementary Figure 6A and B. Each tab of the spreadsheet contains the values and statistics used to generate each panel in the figure. Note: Each line represents the values per patient.

Supplementary Data 8 Source data pertaining to Supplementary Tables 1 - 6. Each tab of the spreadsheet contains the values and statistics used to generate each table.

## Figures and Tables

**Table 1:**
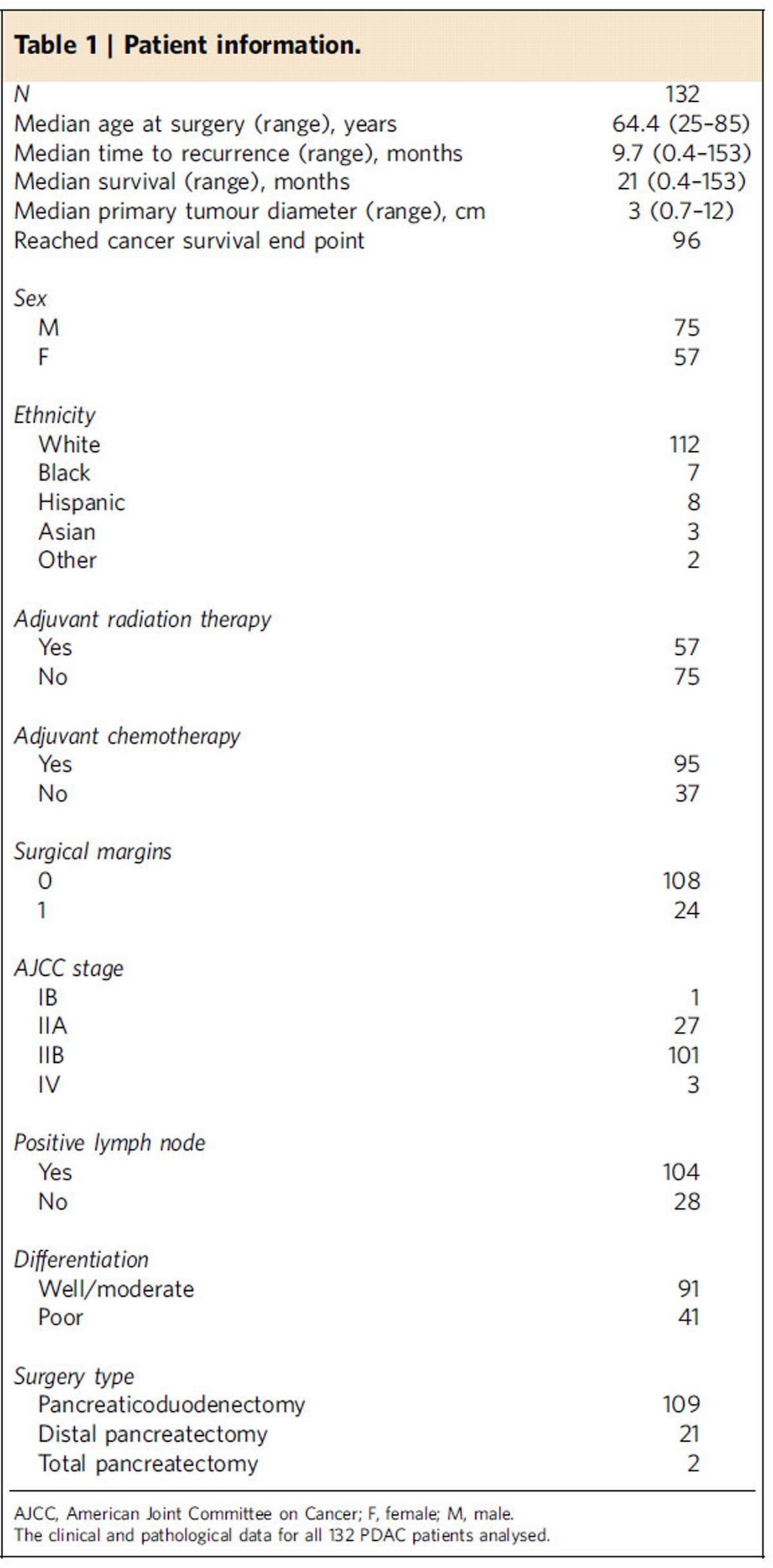
Patient information.

**Table 2:** Univariate and multivariate survival analysis.

	**Univariate**			**Multivariate**	
	**Negative/low**	**Positive/high**	***P*** **value**	***B***	***P*** **value**
Surgical margins	27.5	17.7	0.009	0.75	0.004
Lymph nodes/AJCC stage	44.1	21.8	0.004	1.026	0.001
Adjuvant chemotherapy	15.9	29.4	0.003	−0.944	<0.001
All T cells	18.9	33.1	0.009	−0.51	0.014
Cytotoxic T cells	18.9	27.5	0.004	−0.687	0.002
CD4^+^ T effector cells	21.2	29.4	0.034	−0.447	0.032

AJCC, American Joint Committee on Cancer.

Univariate (Mantel–Cox) and multivariate (Cox regression) survival analyses for each clinical parameter that significantly impacted survival. Surgical margins, lymph nodes and adjuvant chemotherapy were divided into negative and positive groups, while T-cell infiltrations are divided into low and high groups. The median survival times (months) were reported for each of the negative/low and positive/high groups. Of note, as the majority of the patients fall into AJCC stages IIA and IIB (difference in lymph node status, Table 1) the lymph node and AJCC stage parameters were treated as equivalent and combined in the multivariate analysis. The multivariate *B* coefficient and significance are reported, demonstrating all parameters maintain independent effects on survival.

**Table 3:** Sequential Opal multiplex staining protocol.

**Antigen**	**Primary antibody**		**Catalogue number**	**Secondary polymer**		**TSA fluorophore**
	**Concentration**	**Provider**		**Polymer**	**Provider**	
FoxP3	1:50	Abcam	ab20034	Super Picture	Invitrogen	Cy3
CD4	1:20	Thermo	MS-1528	Super Picture	Invitrogen	Cy5
Collagen-I	1:500	AbDSerotec	1310-01	Goat Po-link1	GBI	Coumarin
CD8	1:100	Dako	M7103	Super Picture	Invitrogen	Opal10
Cytokeratin 8	1:50	DSHB	Troma-1	Rat Po-link1	GBI	FITC
αSMA	1:2,000	Dako	M0851	Super Picture	Invitrogen	Cy5.5
CD3	1:500	Dako	A0452	Super Picture	Invitrogen	Opal9
						
*Mouse (*Fig. 2)						
FSP1	1:6,000	Dako	A5114	Super Picture	Invitrogen	680
Cytokeratin 8	1:50	DSHB	Troma-1	Rat-on-Mouse	BioCare	Cy3.5
CD31	1:4,000	SantaCruz	sc-1506	Goat Po-link1	GBI	Cy3
Collagen-I	1:3,000	AbDSerotec	1310-01	Goat Po-link1	GBI	FITC
Ki67	1:200	Thermo	RM-9106-S	Super Picture	Invitrogen	Cy5
αSMA	1:2,000	Dako	M0851	Super Picture	Invitrogen	Coumarin
						
*Human (*Fig. 2)						
CD4	1:25	BioCare	CM153CK	Super Picture	Invitrogen	680
Collagen-I	1:500	AbDSerotec	1310-01	Goat Po-link1	GBI	Coumarin
CD31	1:3,000	SantaCruz	sc-1506	Goat Po-link1	GBI	Cy3
CD8	1:1,000	Dako	M7103	Super Picture	Invitrogen	Cy5
Cytokeratin 8	1:100	DSHB	Troma-1	Rat-on-Mouse	BioCare	Cy3.5
αSMA	1:2,000	Dako	M0851	Super Picture	Invitrogen	FITC
FoxP3	1:25	Abcam	ab20034	Super Picture	Invitrogen	Biotin-Strep 594

αSMA, alpha-smooth muscle actin; FITC, fluorescein isothiocyanate; TSA, tyramide signal amplification.

Each row represents one step in the sequential staining protocol with each individual primary antibody and corresponding secondary HRP polymer and TSA fluorophore. The table is divided into three sections, corresponding to three staining protocols reported. The first section describes the multiplex analysed in the majority of the paper and the second and third sections correspond to the multiplex protocols presented in Fig. 2.
