# Supplementary material for: Spatial computation of intratumoral T cells correlates with survival of patients with pancreatic cancer
Source: Nat Commun. 2017 Apr 27;8:15095. doi: 10.1038/ncomms15095 (PMC5414182; doi:10.1038/ncomms15095)
Supplement: Supplementary Information — Supplementary Figures and Supplementary Tables [file ncomms15095-s9.pdf]

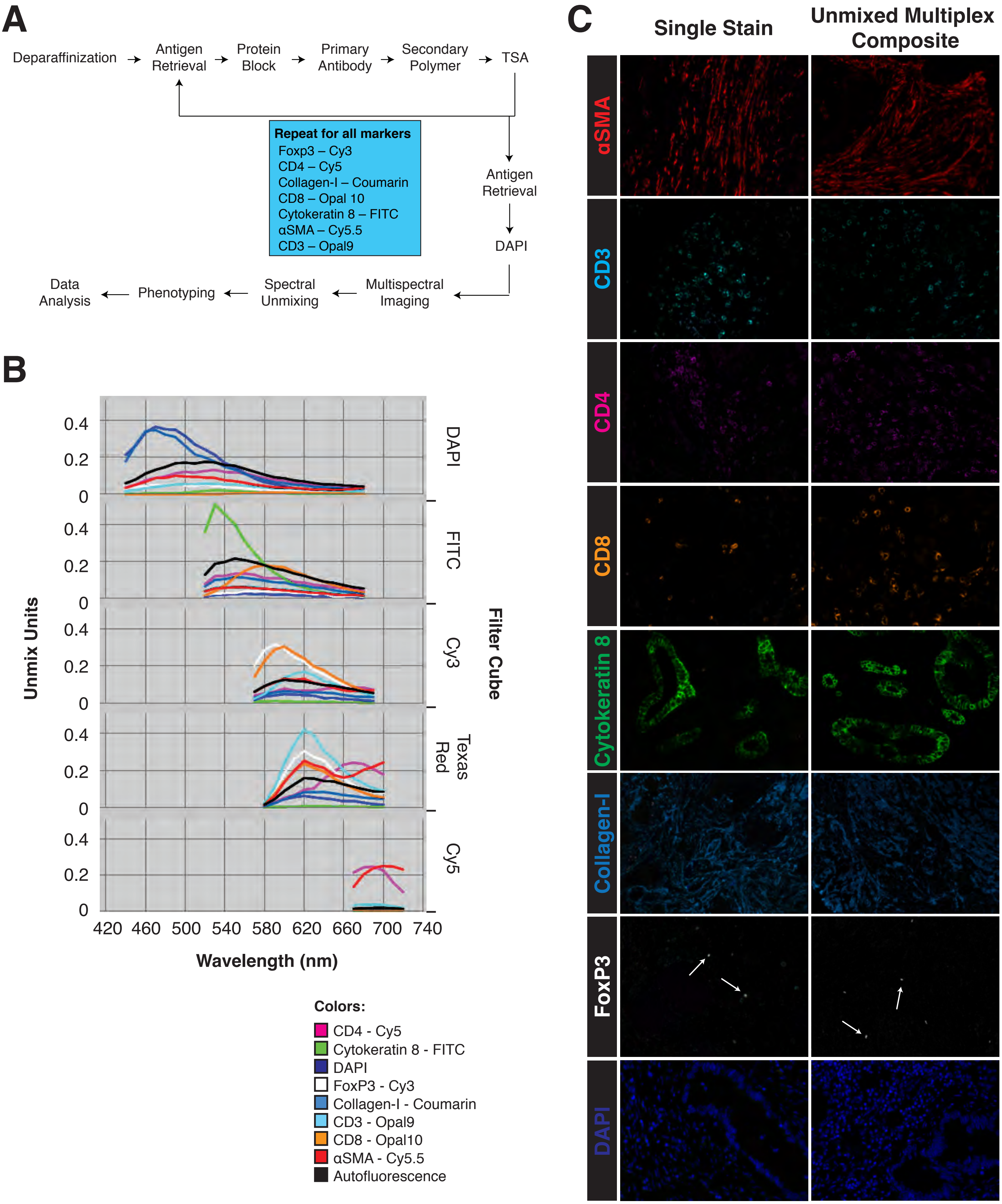

Supplementary Figure 1 – Overview of the eight-color multiplexing protocol. A – Sequential steps of the multiplexing protocol starting with FFPE section deparaffinization and ending with image analysis, including all marker-fluorophore pairs in staining order. **B –** Emission spectrum of all eight marker-fluorophore pairs and the tissue autofluorescence signal used for spectral unmixing. Spectral signatures were captured from single stained controls using multispectral imaging at every 10 nm of wavelength across all filter cubes (DAPI, FITC, Cy3, Texas Red, and Cy5). The unmix units are a function of the dynamic range of the camera. The curves are then normalized to the maximum intensity for each fluorophore (0.5) so they can be presented within the same graph. **C –** Representative images of the eight different markers obtained from single stained controls for each marker compared to single component images obtained from the multiplexed slides after spectral unmixing. The same unmixing algorithm was used for all images, demonstrating a similar staining pattern. Arrows signal FoxP3^{+} nuclei.

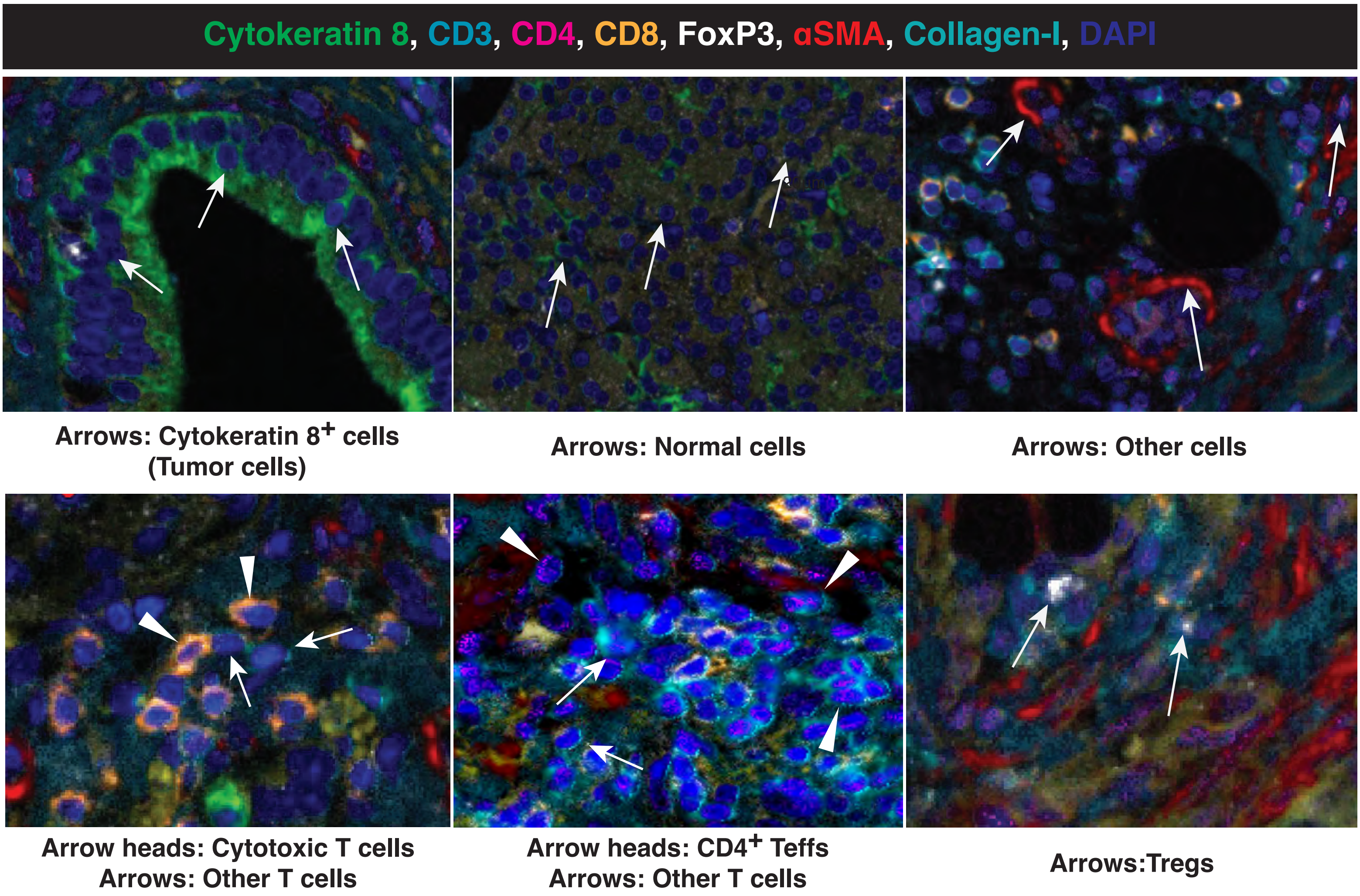

Supplementary Figure 2 – Example staining patterns of defined phenotypes. Representative images of the different cellular phenotypes defined in **Figure 1L**.

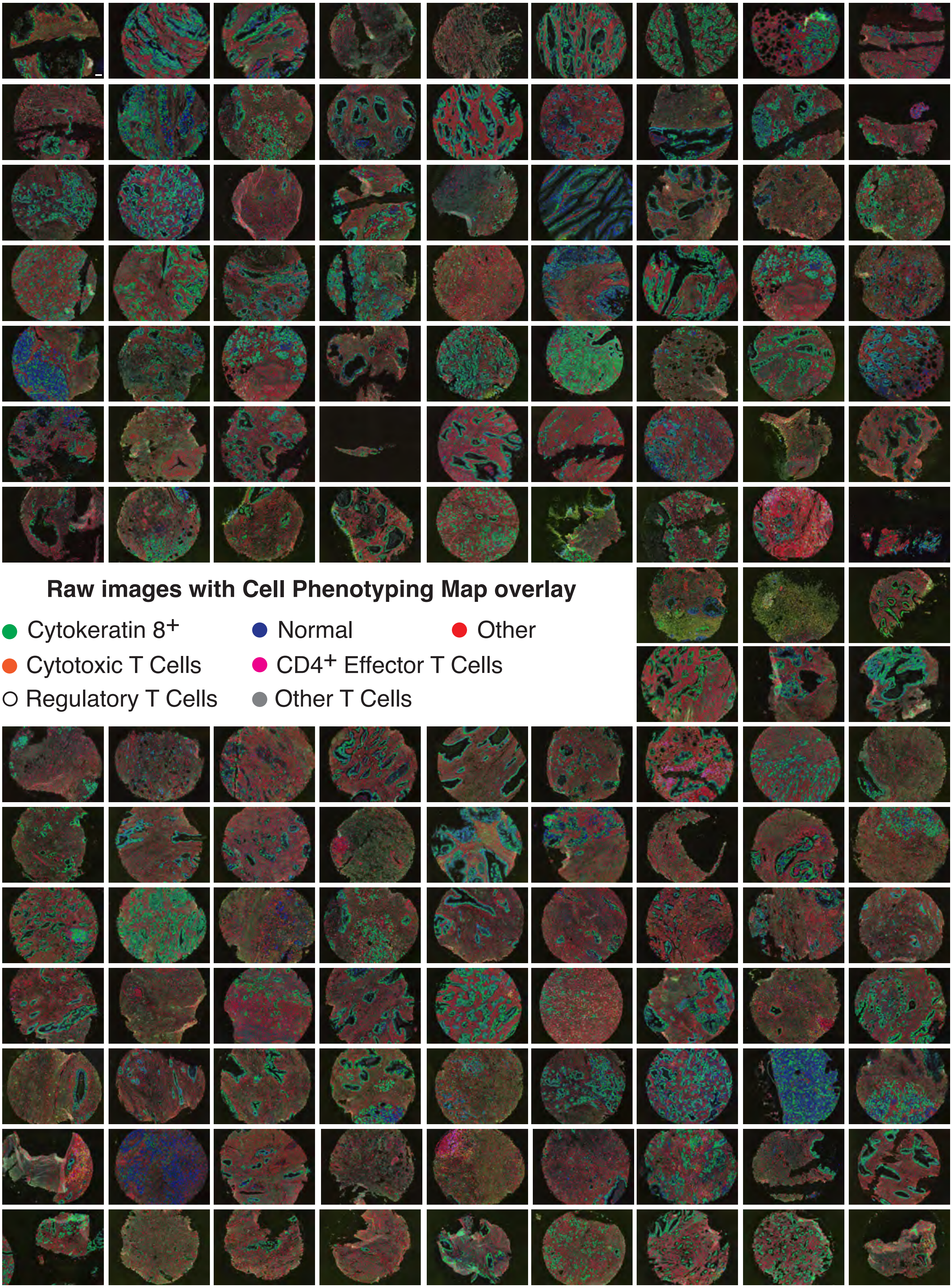

Supplementary Figure 3 – Eight-color multiplex staining of a 132 patient PDAC TMA. Stitched high power (200x) images showing a representative TMA tumor core for each patient. Images displayed are raw images overlaid with the cellular phenotype map displaying all previously defined cell populations (Figure 1L – Tumor/Cytokeratin 8^+ – green, Normal– blue, cytotoxic T cells – orange, $CD4^+$ Effector T cells – magenta, Regulatory T cells – white, Other T cells – grey, and Other – red).

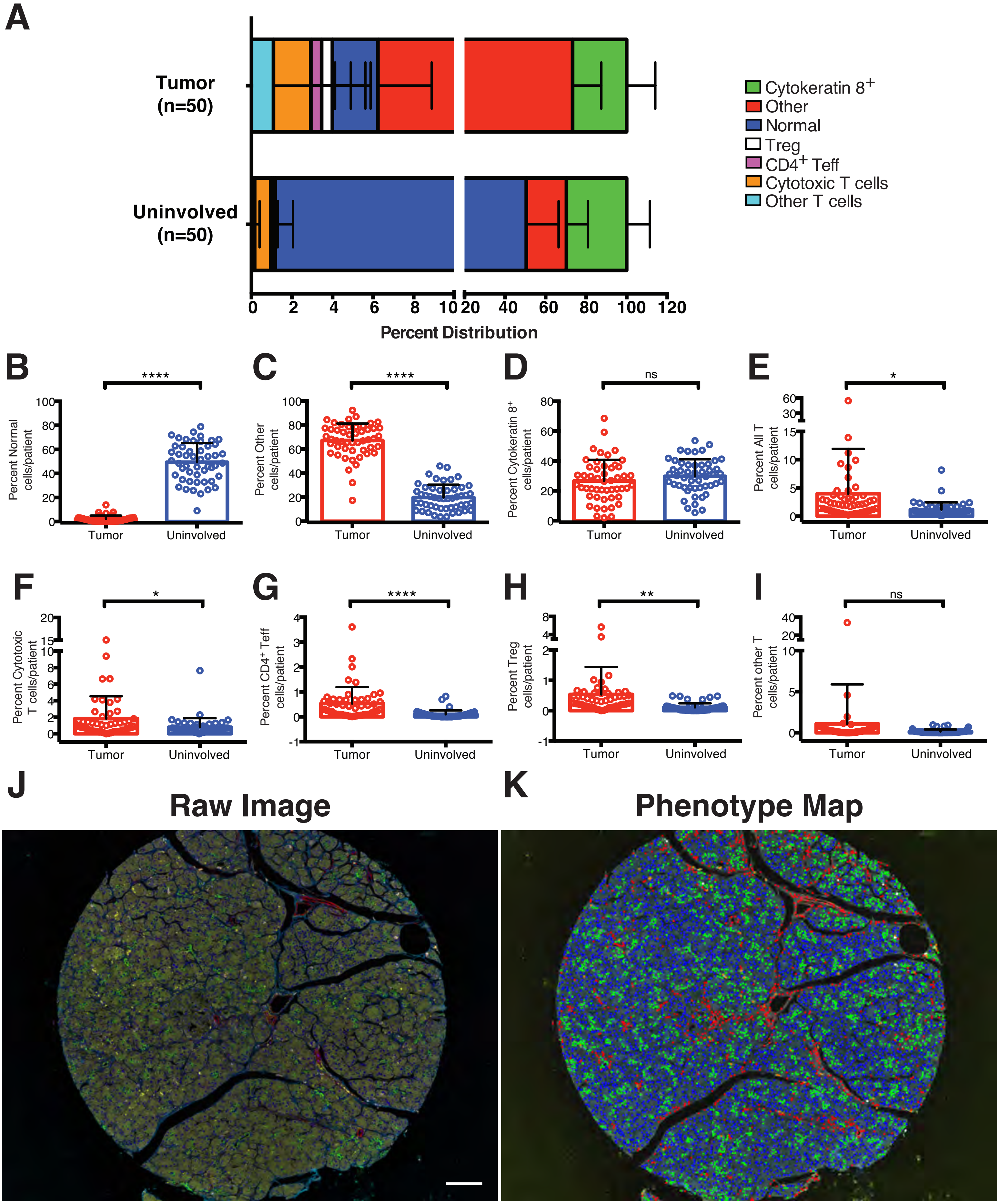

Supplementary Figure 4 – PDAC tumor tissue displays increased infiltration of heterogeneous T cell populations when compared to uninvolved pancreatic tissue. A – Relative distribution of all analyzed cell phenotypes for the 50 patients that have corresponding tumor and uninvolved pancreatic tissue cores. **B-I –** Pairwise comparisons of the percent positive cells per patient for normal cells (**B**), other cells (**C**), cytokeratin 8^+ cells (**D**), all T cells (**E**), cytotoxic T cells (**F**), $CD4^+$ Teff cells (**G**), Treg cells (**H**) and other T cells (**I**) between tumor and uninvolved pancreatic tissue samples. Significance determined by unpaired t-test. **J –** Representative spectrally unmixed (composite) image of an uninvolved pancreatic tissue core. Here the autofluorescence signature has been pseudocolored in yellow to display the unstained acinar cells. **K –** Phenotyping cell map overlay demonstrating the number of cytokeratin 8^+ cells (green dots) and normal cells (blue dots) in the same uninvolved pancreatic tissue core. * $p < 0.05$, ** $p < 0.01$, **** $p < 0.0001$, ns, not significant. Scale bar equals 100 μm.

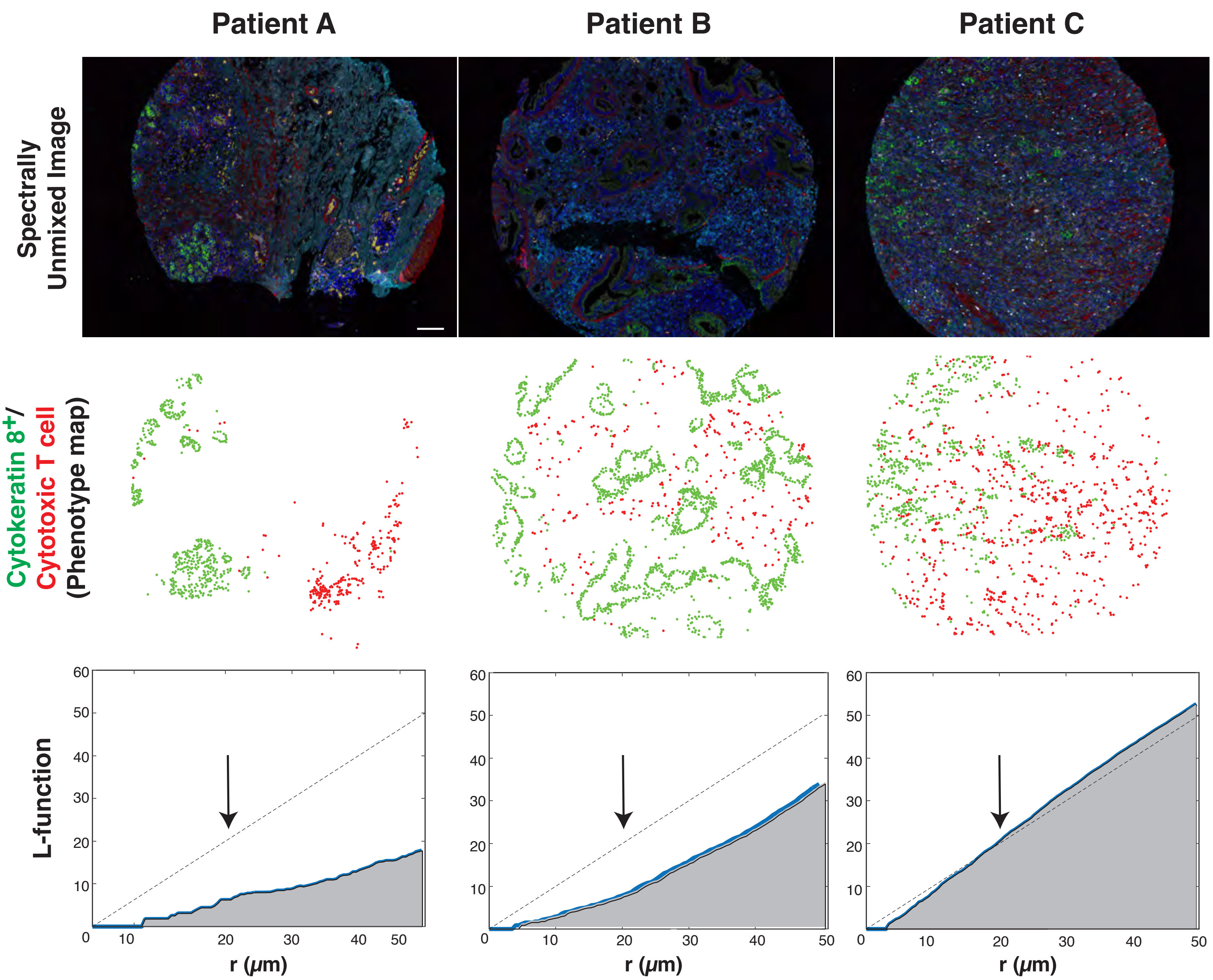

Supplementary Figure 5 – Overview of the generation of AUC values for cytotoxic T cell infiltration relative to cytokeratin 8$^+$ cancer cells. Representative TMA cores from three patients (A, B and C) with differing levels of cytotoxic T cell infiltration into the tumor are shown, with patient A being an example of low infiltration, patient B of medium infiltration and patient C of high infiltration. From top to bottom: TMA cores after spectral unmixing; Cell phenotype map showing only the cytokeratin 8$^+$ cancer cells (green) and cytotoxic T cells (red) in each core; L-function curves representing the spatial distribution of cytotoxic T cells, where the dotted line represents an expected random distribution, the blue line represents the calculated L-function for each core, and the grey area under the L-function curve (AUC) represents the level of infiltration of the cytotoxic T cells relative to cytokeratin 8$^+$ cancer cells. Black arrows point to the differences in the L-function curve and corresponding AUC levels specifically within a radius of 20 μm. Scale bar equals 100 μm.

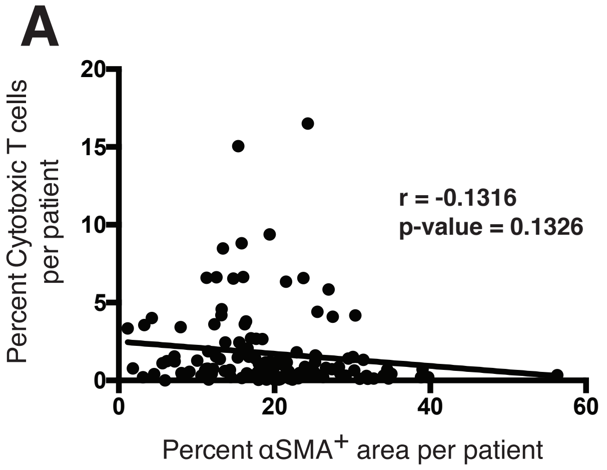

Supplementary Figure 6 - Correlation analysis between the area of αSMA deposition and T cell infiltration. **A -** Correlation analysis between the total area of αSMA deposition and the percentage of Cytotoxic T cell infiltration. **B -** Correlation analysis between the total area of αSMA expression and the percentage of $CD4^+$ Teff infiltration. Pearson correlation coefficient (r) and significance levels (p-value) are presented for each correlation.

	All	High	Low	p Value
**n**	132	66	66	
**Median age at surgery (range), years**	64.4 (25-85)	64.8 (25-81)	64.1 (42-85)	
**Median time to recurrence (range), months**	9.7 (0.4-153)	16 (4.7-137)	7.8 (0.4-153)	
**Median survival (range), months**	21 (0.4-153)	25 (4.7-137)	17 (0.4-153)	
**Median primary tumor diameter (range), cm**	3 (0.7-12)	3 (0.7-12)	3 (0.8-11.5)	
**Reached cancer survival endpoint**	96	44	52	0.171
**Sex**				0.482
M	75	40	35	
F	57	26	31	
**Ethnicity**				0.779
White	112	58	54	
Black	7	2	5	
Hispanic	8	4	4	
Asian	3	1	2	
Other	2	1	1	
**Adjuvant Radiation Therapy**				0.482
yes	57	31	26	
no	75	35	40	
**Adjuvant Chemotherapy**				0.245
yes	95	51	44	
no	37	15	22	
**Surgical Margins**				1.000
0	108	54	54	
1	24	12	12	
**AJCC Stage**				0.144
IB	1	0	1	
IIA	27	16	11	
IIB	101	47	54	
IV	3	3	0	
**Positive Lymph Node**				0.524
yes	104	50	54	
no	28	16	12	
**Differentiation**				0.452
Well/Moderate	91	48	43	
Poor	41	18	23	
**Surgery Type**				0.296
Pancreaticoduodenectomy	109	55	54	
Distal Pancreatectomy	21	9	12	
Total Pancreatectomy	2	2	0	

Supplementary Table 1 – Patient information separated by All T cells

Clinical and pathological data for all patients and patients divided by the high and low total T cells groups as separated by the median percent of all T cells out of total cells per patient. P-values are calculated using the Chi-square analysis comparing the differences between the High and Low groups.

	All	High	Low	p Value
**N**	132	66	66	
**Median age at surgery (range), years**	64.4 (25-85)	65.2 (25-81)	63.5 (40-85)	
**Median time to recurrence (range), months**	9.7 (0.4-153)	16 (4.7-153)	8.1 (0.4-129)	
**Median survival (range), months**	21 (0.4-153)	25 (4.7-153)	17 (0.4-129)	
**Median primary tumor diameter (range), cm**	3 (0.7-12)	3 (0.7-8)	3 (1.5-12)	
**Reached cancer survival endpoint**	96	43	53	0.078
**Sex**				1.000
M	75	37	38	
F	57	29	28	
**Ethnicity**				0.976
White	112	56	56	
Black	7	3	4	
Hispanic	8	4	4	
Asian	3	2	1	
Other	2	1	1	
**Adjuvant Radiation Therapy**				1.000
yes	57	28	29	
no	75	38	37	
**Adjuvant Chemotherapy**				0.699
yes	95	46	49	
no	37	20	17	
**Surgical Margins**				0.822
0	108	55	53	
1	24	11	13	
**AJCC Stage**				0.304
IB	1	0	1	
IIA	27	17	10	
IIB	101	47	54	
IV	3	2	1	
**Positive Lymph Node**				0.287
yes	104	49	55	
no	28	17	11	
**Differentiation**				0.452
Well/Moderate	91	48	43	
Poor	41	18	23	
**Surgery Type**				0.492
Pancreaticoduodenectomy	109	57	52	
Distal Pancreatectomy	21	8	13	
Total Pancreatectomy	2	1	1	

Supplementary Table 2 – Patient information separated by cytotoxic T cells
Clinical and pathological data for all patients and patients divided by the high and low cytotoxic T cell groups as separated by the median percent cytotoxic T cells out of total cells per patient. P-values are calculated using the Chi-square analysis comparing the differences between the High and Low groups.

	All	High	Low	p Value
**n**	132	66	66	
**Median age at surgery (range), years**	64.4 (25-85)	65.2 (40-81)	64.2 (25-85)	
**Median time to recurrence (range), months**	9.7 (0.4-153)	15 (4.7-153)	8 (0.4-137)	
**Median survival (range), months**	21 (0.4-153)	23 (4.7-153)	18 (0.4-137)	
**Median primary tumor diameter (range), cm**	3 (0.7-12)	3 (0.7-12)	3 (0.8-11.5)	
**Reached cancer survival endpoint**	96	43	53	0.078
**Sex**				0.482
M	75	40	35	
F	57	26	31	
**Ethnicity**				0.655
White	112	59	53	
Black	7	2	5	
Hispanic	8	3	5	
Asian	3	1	2	
Other	2	1	1	
**Adjuvant Radiation Therapy**				0.725
yes	57	30	27	
no	75	36	39	
**Adjuvant Chemotherapy**				0.245
yes	95	51	44	
no	37	15	22	
**Surgical Margins**				1.000
0	108	54	54	
1	24	12	12	
**AJCC Stage**				0.144
IB	1	0	1	
IIA	27	16	11	
IIB	101	47	54	
IV	3	3	0	
**Positive Lymph Node**				0.524
yes	104	50	54	
no	28	16	12	
**Differentiation**				0.707
Well/Moderate	91	47	44	
Poor	41	19	22	
**Surgery Type**				0.345
Pancreaticoduodenectomy	109	53	56	
Distal Pancreatectomy	21	11	10	
Total Pancreatectomy	2	2	0	

Supplementary Table 3 – Patient information separated by $CD4^+$ Effector T cells
Clinical and pathological data for all patients and patients divided by the high and low $CD4^+$ Teff cell groups as separated by the median percent $CD4^+$ Teffs out of total cells per patient. P-values are calculated using the Chi-square analysis comparing the differences between the High and Low groups.

	All	High	Low	p Value
**n**	132	66	66	
**Median age at surgery (range), years**	64.4 (25-85)	65.2 (25-81)	63.9 (40-85)	
**Median time to recurrence (range), months**	9.7 (0.4-153)	12 (5.3-137)	8.5 (0.4-153)	
**Median survival (range), months**	21 (0.4-153)	23 (5.3-137)	19 (0.4-153)	
**Median primary tumor diameter (range), cm**	3 (0.7-12)	3 (0.8-12)	3 (0.7-8)	
**Reached cancer survival endpoint**	96	44	52	0.171
**Sex**				0.292
M	75	41	34	
F	57	25	32	
**Ethnicity**				0.414
White	112	59	53	
Black	7	2	5	
Hispanic	8	2	6	
Asian	3	2	1	
Other	2	1	1	
**Adjuvant Radiation Therapy**				0.725
yes	57	30	27	
no	75	36	39	
**Adjuvant Chemotherapy**				0.699
yes	95	49	46	
no	37	17	20	
**Surgical Margins**				0.822
0	108	53	55	
1	24	13	11	
**AJCC Stage**				0.085
IB	1	0	1	
IIA	27	17	10	
IIB	101	46	55	
IV	3	3	0	
**Positive Lymph Node**				0.287
yes	104	49	55	
no	28	17	11	
**Differentiation**				0.452
Well/Moderate	91	43	48	
Poor	41	23	18	
**Surgery Type**				0.972
Pancreaticoduodenectomy	109	55	54	
Distal Pancreatectomy	21	10	11	
Total Pancreatectomy	2	1	1	

Supplementary Table 4 – Patient information separated by Regulatory T cells
Clinical and pathological data for all patients and patients divided by and the high and low Treg cells groups as separated by the median percent Tregs out of total cells per patient. P-values are calculated using the Chi-square analysis comparing the differences between the High and Low groups.

	All	High	Low	p Value
**N**	132	66	66	
**Median age at surgery (range), years**	64.4 (25-85)	64.4 (25-81)	64.4 (45-85)	
**Median time to recurrence (range), months**	9.7 (0.4-153)	12 (4.7-96)	8.9 (0.4-153)	
**Median survival (range), months**	21 (0.4-153)	22 (4.7-96)	21 (0.4-153)	
**Median primary tumor diameter (range), cm**	3 (0.7-12)	3 (0.7-12)	3 (0.8-11.5)	
**Reached cancer survival endpoint**	96	46	50	0.558
**Sex**				0.292
M	75	41	34	
F	57	25	32	
**Ethnicity**				0.908
White	112	57	55	
Black	7	3	4	
Hispanic	8	3	5	
Asian	3	2	1	
Other	2	1	1	
**Adjuvant Radiation Therapy**				0.482
yes	57	31	26	
no	75	35	40	
**Adjuvant Chemotherapy**				0.120
yes	95	52	43	
no	37	14	23	
**Surgical Margins**				0.499
0	108	52	56	
1	24	14	10	
**AJCC Stage**				0.248
IB	1	0	1	
IIA	27	14	13	
IIB	101	49	52	
IV	3	3	0	
**Positive Lymph Node**				1.000
yes	104	52	52	
no	28	14	14	
**Differentiation**				1.000
Well/Moderate	91	46	45	
Poor	41	20	21	
**Surgery Type**				0.774
Pancreaticoduodenectomy	109	56	53	
Distal Pancreatectomy	21	9	12	
Total Pancreatectomy	2	1	1	

Supplementary Table 5 – Patient information separated by Other T cells
Clinical and pathological data for all patients and patients divided by and the high and low other T cell groups as separated by the median percent of other T cells out of total cells per patient. P-values are calculated using the Chi-square analysis comparing the differences between the High and Low groups.

	All	High	Low	p Value
**n**	132	70	62	
**Median age at surgery (range), years**	64.4 (25-85)	64.4 (40-85)	64.5 (25-81)	
**Median time to recurrence (range), months**	9.7 (0.4-153)	11 (4.7-153)	8.7 (0.4-137)	
**Median survival (range), months**	21 (0.4-153)	21 (4.7-153)	21 (0.4-137)	
**Median primary tumor diameter (range), cm**	3 (0.7-12)	3 (0.8-12)	3 (0.8-11.5)	
**Reached cancer survival endpoint**	96	51	45	1.000
**Sex**				1.000
M	75	40	35	
F	57	30	27	
**Ethnicity**				0.657
White	112	62	50	
Black	7	2	5	
Hispanic	8	4	4	
Asian	3	1	2	
Other	2	1	1	
**Adjuvant Radiation Therapy**				1.000
yes	57	30	27	
no	75	40	35	
**Adjuvant Chemotherapy**				0.565
yes	95	52	43	
no	37	18	19	
**Surgical Margins**				0.115
0	108	61	47	
1	24	9	15	
**AJCC Stage**				0.198
IB	1	0	1	
IIA	27	12	15	
IIB	101	55	46	
IV	3	3	0	
**Positive Lymph Node**				0.287
yes	104	58	46	
no	28	12	16	
**Differentiation**				0.189
Well/Moderate	91	52	39	
Poor	41	18	23	
**Surgery Type**				0.260
Pancreaticoduodenectomy	109	59	50	
Distal Pancreatectomy	21	9	12	
Total Pancreatectomy	2	2	0	

Supplementary Table 6 – Patient information separated by the $CD4^+$ Effector/Regulatory T cell ratio
Clinical and pathological data for all patients and patients divided by the high and low $CD4^+$ Teff/Treg ratio groups as separated by the median ratio per patient. P-values are calculated using the Chi-square analysis comparing the differences between the High and Low ratio groups.
